# UV Activation of Persulfate for Removal of Penicillin G Antibiotics in Aqueous Solution

**DOI:** 10.1155/2017/3519487

**Published:** 2017-08-08

**Authors:** Samira Norzaee, Edris Bazrafshan, Babak Djahed, Ferdos Kord Mostafapour, Razieh Khaksefidi

**Affiliations:** ^1^Department of Environmental Health Engineering, Iranshahr University of Medical Sciences, Iranshahr, Iran; ^2^Torbat Heydariyeh University of Medical Sciences, Torbat Heydariyeh, Iran; ^3^Health Promotion Research Center, Faculty of Health, Zahedan University of Medical Sciences, Zahedan, Iran; ^4^Department of Environmental Health Engineering, Faculty of Health, Zahedan University of Medical Sciences, Zahedan, Iran

## Abstract

Penicillin G (PG) is one of the most widely consumed antibiotics around the world. Release of PG in environment may lead to contamination of water resources. The aim of the present work is to assess feasibility of applying UV-activated persulfate process in removal of PG from aquatic environments. The study examined the effect of pH (3–11), persulfate initial concentration (0.5–3 mM), reaction time (15–90 minutes), and initial concentration of PG (0.02–0.14 mM) on PG decomposition. Also, the pseudo-first-order kinetic model was used for kinetic analysis of PG removal. The results indicated that UV-activated persulfate process can effectively eliminate PG from water. The highest PG removal efficiency was obtained as 94.28% at pH 5, and the decomposition percentage was raised by increasing persulfate dose from 0.5 to 3 mM and the reaction time from 15 to 90 minutes. Besides, the removal efficiency decreased through increasing the initial concentration of PG. UV-activated persulfate process effectively decomposes PG and eliminates it from water.

## 1. Introduction

Pharmaceutical compounds may not differ from other chemicals such as pesticides and herbicides in terms of environmental hazards. These compounds enter the aquatic environment through wastewater of the pharmaceutical industry as well as the final consumer in a metabolized or nonmetabolized form [1]. Among the pharmaceutical compounds, antibiotics have an important contribution to the environmental pollution due to high consumption in medicine and veterinary [2]. Considering 30–90 percent of nonmetabolized antibiotics in human and animal body released into the environment through urine and feces in the form of active ingredients, microbial resistance would be expected in our environment [3].

**Figure 1:**
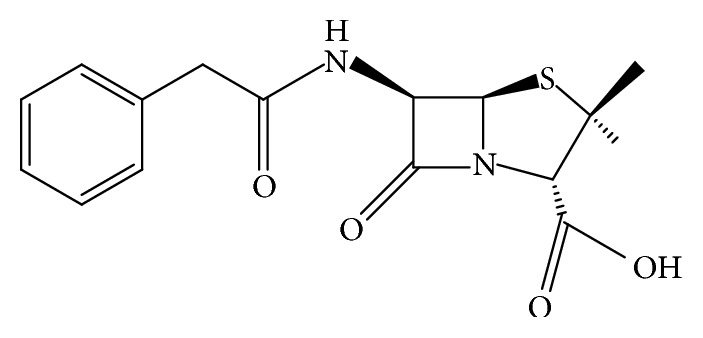
Structural formula of Penicillin G.

Penicillin G (PG) is one of commonly used antibiotics (Figure 1) that is utilized for the treatment of various human bacterial infections [4, 5]. A part of PG may pass through aerobic and anaerobic treatment units of wastewater treatment plants and some studies indicated that the concentrations of PG in raw and treated wastewater were 153 and 1.68 mg L^−1^, respectively [6]. Hence, considering the health risk related to the entering of the antibiotics to the environment, they require approaching an allowable discharge limit before being disposed into the environment and aquatic ecosystems.

Recent studies applied various methods for removing pharmaceutical compounds such as reverse osmosis [7], adsorption on activated carbon [8], and ozonation [9] as well as advanced oxidation systems (AOP) such as Fenton or Photo-Fenton [10], ultrasound [11], preoxidation using UV lamps [12], and photocatalysis with TiO_2_ [13]. Due to some troubles in using the aforementioned processes, advanced oxidation system has gained interest in recent years [14].

AOP technology mainly works based on producing reactive species resulting from decomposition of oxidants such as persulfate (S_2_O_8_^−2^); this compound through producing SO_4_^−∙^ radical (a very powerful oxidant with oxidation-reduction potentials of 2.5–3.1 V) could effectively decompose organic materials [15]. Sulfate radical is a unique oxidant because of its characteristics, including its high stability compared to hydroxyl radicals, high reaction rate, and effective influences on organic matters [16, 17]. Overall, SO_4_^−∙^ directly changes the electron produced from organic compounds into organic radical cations [18]. The efficiency of PS alone has been less to decompose organic compounds; in addition, it needs to be activated in order to accelerate the process of sulfate radical production. PS is usually activated by heat, UV, and transition metals [17]. UV activation, among these methods, not only is an effective way of water disinfection, but also can be considered as a form of energy for persulfate activation [19]. Several studies utilized this method to eliminate compounds such as acid blue 113 [20], tetramethylammonium hydroxide [19], and sulfamethazine [21]. PS activation by UV radiation produces two moles of sulfate radical from a mole of sulfate according to

(1) $$\begin{array}{c} S_{2}{O_{8}}^{2-}\overset{UV}{\to }{{2SO}_{4}}^{∙} \end{array}$$

The aim of this study is to examine the feasibility of UV/PS process in PG decomposition from aquatic solutions. Also, the effects of pH, initial concentration of PS, reaction time, and initial concentration of PG on PG decomposition efficiency were investigated through UV/PS process.

## 2. Materials and Methods

### 2.1. Apparatus and Materials

Sodium persulfate (Na_2_S_2_O_8_) and Penicillin G sodium were purchased from Sigma-Aldrich Chemical Company. Mercury (II) sulfate, potassium dichromate, silver sulfate, sulfuric acid, and sodium hydroxide were prepared from Merck Company. A 30 W ultraviolet lamp (Philips, with maximum emission at 254 nm) with 9 cm length and diameter of 28 mm was used in the experiments. Concentration of PG and COD was determined through a UV-Vis spectrophotometer (HACH, DR 5000).

### 2.2. Methodology

**Figure 2:**
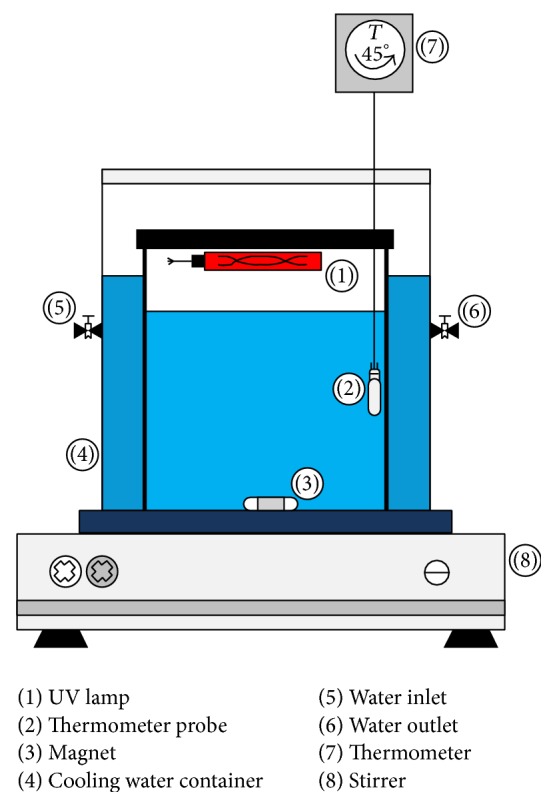
Setup for experiments.

The study was conducted through batch mood experiment. A Plexiglas square shape reactor (total volume of 2 liters) was utilized in the experiments (Figure 2); and it was equipped with a UVC lamp that was installed 7 cm above the sample surface, the intensity of UV irradiation was 950 *μ*w/m^2^.

PG and PS stock solutions were prepared daily using deionized water. To make solutions with different concentrations, the stock solution was diluted using deionized water. Also, the reactor was covered with aluminum sheets for the possibility of UV light reflection output. Besides, temperature of the solution was adjusted to 25 ± 3°C through adjusting the cooling water flow and it was monitored by a thermometer continuously. The effect of several parameters including pH (3, 5, 7, 9, and 11), contact time (15, 30, 60, and 90 minutes), initial concentration of PS (0.5, 1, 1.5, 2, 2.5, and 3 mM), and different concentrations of PG (0.02–0.14 mM) on PG decomposition was also investigated. For this purpose, a parameter was changed and three parameters were kept constant in each examination period. pH of the solution was adjusted using HCl and NaOH; and then, it was transferred to the reactor after adding PS. To keep the solution homogeneous during reaction time, the magnetic stirrer with speed of 100 rpm was used. Next, in order to activate PS, it was radiated by UV emission. Then, 10 ml of the solution was taken from the upper part of the reactor as the sample at the defined time intervals. To determine the remaining PG in solution, the concentration of this substance at a wavelength of 290 nm was reported; also, in order to determine the concentration of the remaining PG along with the intermediate compounds resulting from decomposition of PG, the amount of COD was measured in each experiment. Besides, for surveying the decomposition of PG by UV alone, the concentration of 0.02 mM of PG was irradiated with UV at various contact times; moreover, to examine the effect of PS alone, in darkness condition, the degradation of PG (0.02 mM) was studied in the presence of PS (2 mM).

### 2.3. Kinetics of the Reaction

Kinetics of PG decomposition by UV/PS were investigated through pseudo-first-order equation (see (2)). Most studies indicated that oxidation of the organic pollutants by PS follows the pseudo-first-order equation [22, 23].

(2) $$\begin{array}{c} -\frac{dPG}{dt}=k\left[\;PG\;\right]. \end{array}$$

Equation (2) can be rewritten as follows [24]:

(3) $$\begin{array}{c} \ln\frac{C_{i}}{C_{t}}=kt, \end{array}$$

where *C*_*i*_ (mg L^−1^) is PG concentration (mg L^−1^) at time *t*, *k* is the reaction rate (min^−1^), and *t* (min) represents the reaction time.

## 3. Results and Discussion

### 3.1. Effect of pH

**Figure 3:**
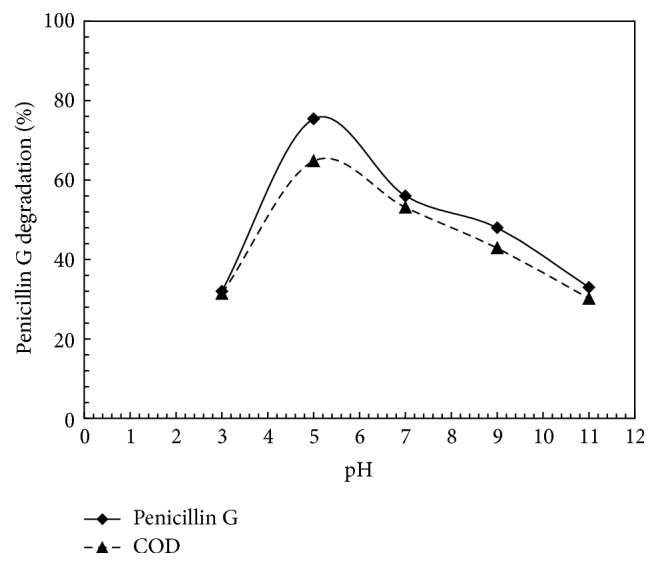
Effect of pH on PG and COD degradation by UV-activated persulfate. PG = 0.02 mM, time = 60 min, and persulfate = 2 mM.

The removing efficiency of PG in various pH values (3, 5, 7, 9, and 11) was investigated in order to evaluate the effect of pH on PG removal in UV/PS process. For this purpose, PS initial concentration (2 mM), PG initial concentration (0.02 mM), and reaction time (60 minutes) were kept constant. The results are shown in Figure 3. According to Figure 3, the highest PG removal rate (75.4%) is related to pH 5. The results revealed that increasing of the pH from 3 to 5 intensifies PG removal efficiency, while increasing the pH from 5 to 11 may decrease the efficiency up to about 53.2%. Also, as seen in Figure 3, COD removal at pH of 3, 5, 7, 9, and 11 was 31.4, 64.8, 53.1, 42.9, and 30.2 percent, respectively, where the highest removal rate was observed at pH 5. As it is known, one of the most influential parameters of chemical processes, especially in advanced oxidation processes, is the pH of the solution, which the rate of chemical reactions relies on. According to the results of the conducted studies, pH directly affects radical production [25]. According to the results of this study, the efficiency of PG removal reduces as pH increases. Hydroxyl radicals (HO^•^) dominate at pH 11 in accordance with (4). Based on these results, decrease in efficiency may be attributed to the reaction between hydroxyl radical and sulfate radical, which will lead to the consumption of both radicals (see (5)) [26].

(4) SO4−∙+OH−⟶SO42−+OH∙Alkaline  pH

(5) SO4−∙+OH∙⟶HSO4−+0.5O2

(6) OH∙+OH−⟶O∙−+H2O

Moreover, the concentration of hydroxyl ions at high pH increases scavenging the hydroxyl radical (see (6)) [16]. In other words, at higher pH values, the presence of high amounts of OH^•^ radicals causes radical–radical reactions and then leads to the consequent deactivation of OH^•^ radicals [27]. In addition, at low pH, SO_4_^−∙^ is dominant. However, in a highly acidic condition, SO_4_^−∙^ is scavenged by the SO_4_^−∙^ itself and it decreases the removal efficiency [28]:

(7) $$\begin{array}{c} {{SO}_{4}}^{-∙}+{{SO}_{4}}^{-∙}⟶{S_{2}O_{8}}^{2-} \end{array}$$

At a pH close to neutral, SO_4_^−∙^ and OH^∙^ are dominant and they decompose PG according to the following [29]:

(8) 2SO4−•+PG⟶PG•+products

(9) SO4−∙+PG∙⟶chain  termination

Therefore, at a pH close to neutral, the sulfate radical demonstrated the best efficiency of decomposing pollutants.

### 3.2. Effect of Persulfate Concentration

One of the effective parameters of advanced oxidization processes is the concentration of the oxidizer. Among the different oxidizers, sulfate radical is capable of decomposing resistant organic compounds. It directly and indirectly decomposes organic compounds. In direct method, sulfate radical decomposes the pollutant directly (see (9)); and in indirect method, it highly decomposes the pollutants through producing hydroxyl radicals [29]:

(10) SO4−∙+H2O⟶OH∙+SO42−+H+

(11) OH∙+PEN  G⟶products

**Figure 4:**
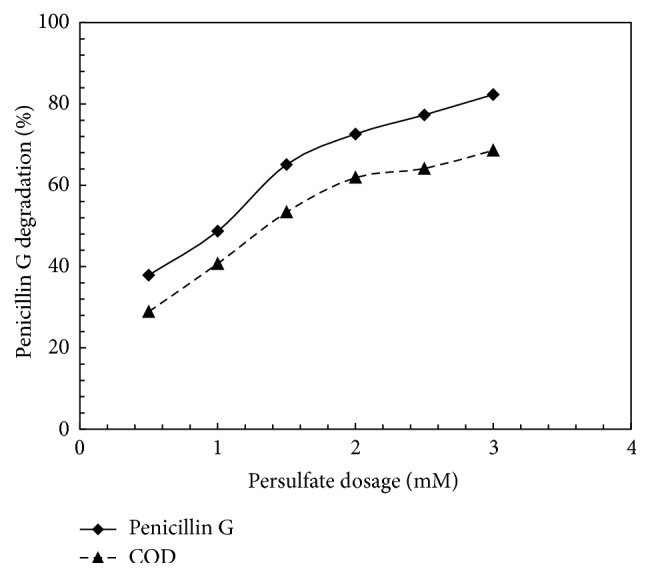
Effect of persulfate concentration on PG and COD degradation by UV-activated persulfate. PG = 0.02 mM, time = 60 min, and pH = 5.

In order to survey the effect of initial concentration of PS on PG removal efficiency, various initial concentrations of PS (0.5, 1, and 1.5 and 2, 2.5, and 3 mM) were examined. For this purpose, PG concentration (0.02 mM) and reaction time (60 minutes) were constant and pH was adjusted to 5. According to Figure 4, the results revealed that, through increasing PS concentration from 0.5 to 3 mM, PG removal rate increases. Then, by a further increase in concentration of PS, the removal efficiency increases at a low slope. The highest efficiency was also obtained at 82.3% at a PS concentration of 3 mM. As seen in Figure 4, COD removal rate at concentrations of 0.5, 1, 1.5, 2, 2.5, and 3 mM of PS was obtained at 28.9, 40.7, 53.4, 61.9, 64.1, and 70.6 percent, respectively, where the highest removal rate was observed at a concentration of 3 mM that it was similar to the removal pattern of PS concentration. The increasing of COD and PG removal efficiency through increasing of PS concentration is probably due to increased production of SO_4_^−∙^. However, results of some studies indicated that increasing of PS concentration more than a certain amount not only may not increase the decomposition of pollutants but also is a factor of abstraction and consumption of sulfate radical and, consequently, decreasing of the decomposition efficiency [26]:

(12) $$\begin{array}{c} {{SO}_{4}}^{-∙}+S_{2}{O_{8}}^{2-}⟶{{SO}_{2}}^{2-}+S_{2}{O_{8}}^{-∙} \end{array}$$

Also, high concentrations of PS under UV radiation can produce more H^+^ and this results in a reduction of pH influencing removal efficiency [30]. However, in this study, the highest utilized persulfate dose (3 mM) has not reached the critical level to reduce the rate of decomposing PG, and for this reason the PS inhibitory effect was not observed. Therefore, in current survey, it can be concluded that the concentration of 3 mM was as the desired concentration of PS.

### 3.3. Effect of Penicillin G Concentration and Reaction Time

**Figure 5:**
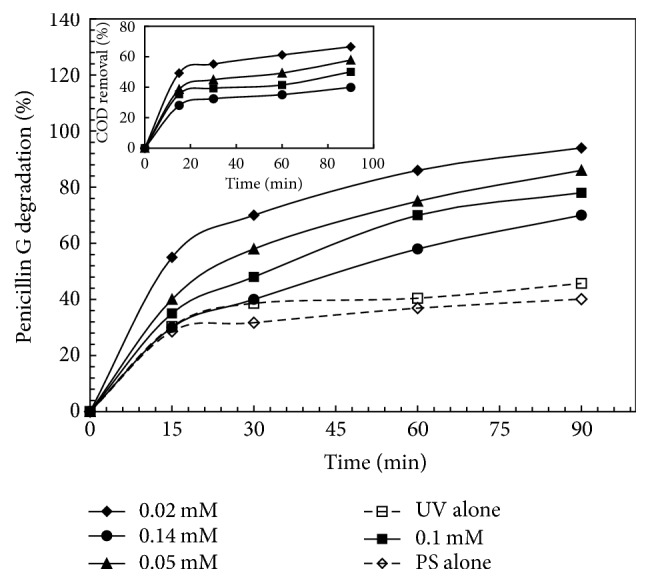
Effect of initial concentration of PG and reaction time on PG and COD degradation by processes of UV-activated persulfate, PS alone, and UV alone. Persulfate = 3 mM and pH = 5.

In order to investigate the effect of the initial concentration of PG as well as reaction time on UV/PS process, the PG removal efficiency in various initial concentrations of PG (0.02–0.14 mM) and at 4 different reaction times (15–90 minutes) was investigated; it is worth mentioning that the PS concentration and pH were fixed at 3 mM and 5, respectively. According to the obtained results (Figure 5), by increasing of reaction time from 15 to 30 minutes, the removal rate increased at all understudied concentrations so that, at an initial concentration of 0.02 mM of PG, the removal rate increased from 60.44 to 80.11 percent. However, by increasing the reaction time to 90 minutes, the removal efficiency curve is augmented with a low slope. Kordkandi and Forouzesh [23] in a study concluded that, during time increasing, the lower slope of the PG removal curve is attributed to the decreasing of the PG concentration; this situation leads to lower probable contact of pollutant with radicals. As shown in Figure 5, PG removal rate declined by increasing of PG initial concentration; furthermore, the COD removal rates at 90 min for concentrations of 0.02, 0.05, 0.1, and 0.14 mM were obtained at 66.5, 57.8, 50.1, and 39.9 percent, respectively. Since forming free produced radicals is constant for a defined concentration of PS, therefore, available sulfate radicals for decomposing of high concentrations of PG may be inadequate; this condition leads to decrease of PG removal by increasing of initial PG concentration. In addition, oxidation of PG intensifies concentration of intermediate compounds; these produced intermediate compounds consume the radicals; this would reduce PG decomposition through free radicals [31–33]. Also, regarding the short lifetime of the produced free radicals such as sulfate radical and hydroxyl radical, which are about 4 seconds and 20 nanoseconds, respectively, they may react with PG molecules immediately after production and before reaction with intermediate compounds. For this reason, lower concentrations of PG lead to less creation of intermediate compounds; hence, the removal rate is enhanced, whereas, with increasing PG concentrations where further intermediate compounds are formed, the removal efficiency is dropped [15]. Figure 5 also represents the results of comparing PG removal rates through the processes of UV/PS, PS alone, and UV alone. According to the obtained results, UV/PS process is generally a more effective method than the other two methods in removing of PG. As known, the main mechanism in UV alone is direct UV radiation that it degrades the pollutants directly, while, at PS alone, the dominant mechanism is oxidation using PS. Although PS is a strong oxidant with a redox potential of 2.01 V, oxidation by the PS is often a slow process [19]. Similar results were reported where degradation of various contaminants was distinctly promoted in the UV/PS system compared with UV alone [30, 34]. These promotions were related to the generation of SO_4_^−∙^ through the combination of chemical oxidation and photodegradation, which has the effect of synergistic enhancement [34]. PS in the presence of UV can be converted into two SO_4_^−∙^ molecules (see (1)) [21]. Then, according to reactions of (7) and (8), it oxidizes PG.

**Figure 6:**
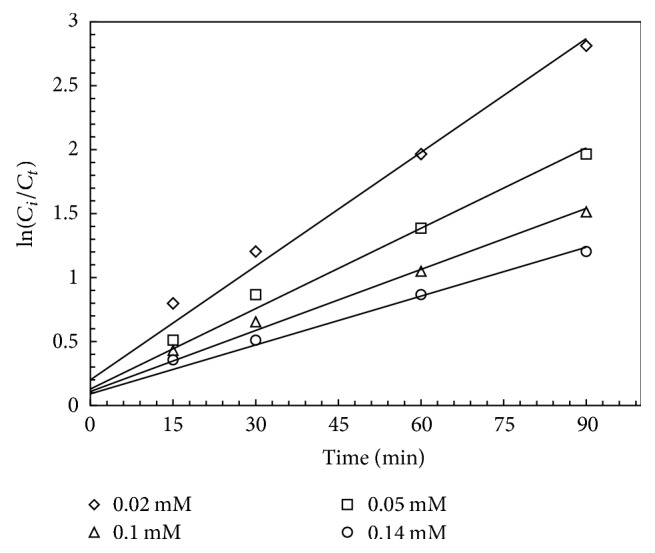
ln⁡(*C*_*i*_/*C*_*t*_) versus reaction time.

Besides, in order to study the PG decomposition kinetics using UV/PS process, the pseudo-first-order model was utilized which is usually used to describe the decomposition of various organic compounds, especially antibiotics [30, 35]. In the pseudo-first-order kinetic model, slope of ln⁡(*C*_*i*_/*C*_*t*_) against the reaction time is equal to *K* constant (Figure 6). Also, the rate constant of the reaction (*K*) and the regression coefficients of *R*^2^ were indicated in Table 1.

## 4. Conclusion

The current research showed that the PG decomposition efficiency through UV/PS process is influenced by parameters such as pH, initial concentration of PS, initial concentration of PG, and reaction time. According to the obtained results, applying UV is an effective method for activation of PS in order to remove PG. Besides, we found that the highest removal efficiency of 94.28 percent was obtained at pH 5, a PS concentration of 3 mM, reaction time of 90 minutes, and PG concentration of 0.02 mM. Also, it was revealed that, by increasing initial concentration of PG from 0.02 to 0.14 mM, the constant reaction rate (*k*) reduced from 0.0296 to 0.0127 min^−1^. Besides, the results of COD removal demonstrated that the UV/PS process may be effectively used for PG decomposition. Finally, it was found that UV/PS process can be considered as an effective method for removing PG from pharmaceutical industry as well as hospital wastewater.

## Figures and Tables

**Table 1:** Rate constant (*K*) and correlation coefficient (*R*^2^) for different initial concentrations of PG.

*C*_*t*_ (mM)	*K* (min^−1^)	*R* ^2^
0.02	0.0296	0.9903
0.05	0.0259	0.9907
0.1	0.0171	0.9802
0.14	0.0127	0.985
